# Facts Connected with the Recent Spread of Cholera

**Published:** 1866-09

**Authors:** 


### Facts connected with the recent spread of Cholera.

August 15th.—The five months that have elapsed since these lectures were delivered have added somewhat to the great cholera record; and though the facts are probably familiar to most of the readers of the Record, it may be well to bring them together and examine their bearings.

The steamship *England*, for New York, left Liverpool on the 28th of March, touching at Queenstown, Ireland, the 29th, having, on leaving the latter port, 1185 steerage passengers, mostly German and Irish, 17 saloon passengers, and a crew of 122. April 1st, a German boy in the steerage was attacked with cholera and died. On the 3d the vessel experienced very heavy weather, and the hatches were battened down for two nights. When opened, it was found that another case of cholera had occurred, "which proved fatal in four hours; after this the disease spread rapidly." When she put into Halifax, on the 9th, the surgeon, Dr. McCullogh, reported 160 cases and 46 deaths. On the morning of the 11th the sick were removed from the *England* and placed on board the *Pyramus*, now converted into a hospital-ship, and about the same time the healthy passengers of the steerage were placed in encampments under tents. On the evening of the 11th it was found that forty persons had died in the last twelve hours, that there were then 100 sick, and that new cases were occurring every hour. On the 13th it was reported that the deaths were twenty-five a day, three or four of which were in the encampments, the others on board the *Pyramus*. The saloon passengers and the crew remained on board the *England*, but among them there was no sickness. Indeed, there was no cholera among the saloon passengers at any time, but before arriving at Halifax six of the crew had died of it. Many relatives of the sick followed the patients to the *Pyramus*, so that the inmates of this ship were about 400; among these there was a great deal of sickness. Diarrhœa prevailed extensively in the encampment, but there were only a few cases of full cholera.

On the 18th, the *England* "having been thoroughly scraped, scrubbed, and fumigated with chloride of lime and sulphuric acid," the healthy passengers were permitted to return to her, the greatest care being exercised "to prevent any but those in perfect health from being taken on board." The *England* steamed out of the harbor on the afternoon of the 18th of April, having been in port nine days, for New York, with 875 steerage and 16 saloon passengers, and a crew of 116. One saloon passenger had died of apoplexy.

Nearly a month later, that is, the 17th of May, the survivors of those that remained on board the *Pyramus*, forty-two in number, were sent to New York by the steamer *Louisa Moore*. Both these ships arrived in New York harbor without any more sickness. All the deaths among the passengers and crew up to the 18th were 250.

Seventeen men were sent down from Halifax to clean the ship *England* on the 11th. On the 13th one of these men was attacked with cholera and died in about four hours. He had volunteered to assist Dr. Garvie to remove the dead bodies from the *England*. But that this humane act could have had no influence in producing the disease is evident from the fact that the work lasted three hours, and he fell sick immediately after it was completed. He had fallen from the vessel into the water the day before, and was much exhausted before he could be rescued.

A pilot, with one other man in his boat, spoke the *England* when outside the harbor of Halifax, and, learned she was infected with cholera, he refused to go on board, but took a line from the ship and was towed after her, giving directions from his boat. It is said that "the stench from the ship was intolerable." This was on the 9th. The pilot was intoxicated for two days after, and Dr. Jennings found him at 7 P. M. on the 11th in collapse. He drank some gallons of water in which bicarbonate of soda was dissolved, and vomited and purged as much. He passed through the collapse, and died the fourth day after in the typhoid stage. This man had a wife and five children. His wife was his only nurse and she escaped the disease, but the five children all had it, and two of them died. The pilot lived, not in Halifax, but at a place called Portuguese Cove.

Dr. Slayter, Health Officer, boarded the *England* on the 9th, and nobly volunteered to assist the physicians of the ship in taking care of the sick. He fell a victim to his generous impulses. He was attacked on the afternoon of the 16th and died the next morning. Dr. Gossip, Dr. Garvie, and Mr. F. F. Garvie, whom I am proud to recognize as a student of the College of Physicians and Surgeons, with equally noble purpose volunteered for similar duty and escaped the disease; as did the ship's surgeon and his assistant, and Dr. Voegles, a saloon passenger, who, on the breaking out of the disease, offered his services to the ship's surgeon, and continued with the sick till the *England* sailed from Halifax.

The only cases that occurred in Halifax, among persons that had not been in intercourse with the *England*, were in one family living near Freshwater Bridge. Dr. Woodill says that the father admitted that underclothes had been made for the child first attacked out of material picked up on the shore, "supposed to be from the *England*." Dr. Jennings says: "The child was lying on a mattress which I have heard was made from some of the bedding thrown from the ship *England*." The father would not admit this to Dr. Woodill, but his mode of answering questions relating to it left on this gentleman's mind the impression that there was something important in the matter which he wished to conceal. This family, consisting of father, mother, and two young children, were all sent to the City Hospital. The child first attacked had cholera symptoms on the 22d of April, having had diarrhœa for some days before, and died soon after being removed to the hospital. At that time the other child had diarrhœa, which was not controlled till there were some symptoms of collapse; she recovered. The mother also had diarrhœa, which was controlled. On the 25th, however, it recurred. On the morning of that day she washed some clothes soiled by the child then sick. In the afternoon she drank largely of water intended for washing, and was almost immediately seized with diarrhœa. She died on the 30th. The father had two attacks of diarrhœa while in the hospital, but did not otherwise suffer. No other cases occurred in Halifax or the neighborhood, unless a severe diarrhœa affecting the daughter of the man who was the pilot's companion in the boat should be added to the catalogue. She had washed her father's clothes after he returned from the ship, and fell sick the next day.

One fact not often noticed after cholera is recorded of cases at Halifax. The mother at the hospital, seized on the 25th, was recovering on the morning of the 27th. The blueness disappeared from all parts of the body except the end of the nose. "On the 28th, *gangrene* of the nose set in, and on the 29th sloughing had commenced. She lingered in a very low condition, the pulse gradually failing, until the 30th." Among the patients treated on the *England*, seven, when recovering from cholera, but still debilitated, had inflammation of the feet. "Two of these recovered perfectly; in the other five gangrene supervened, in two instances of the feet, in three of the toes only." These patients were still under treatment at the City Hospital on the 6th of June.

This account I have condensed from the official report of Dr. Gossip, who succeeded the lamented Slayter as Health Officer. I

should add one other fact stated by Dr. Gossip, viz: that twelve of the steerage passengers escaped from the encampment, though it was on an island. We learn from other sources that one of these was subsequently attacked with cholera in Portland, Me.

Here we find a ship frightfully crowded, the hatches battened down upon 1184 breathing mortals, for two days, after one case of cholera had occurred among them. "After this the disease spread rapidly!" Did cholera ever fail to spread rapidly under such circumstances? One hundred and sixty cases in four days! In port, her living or rather dying freight is removed, the well separated from the sick, and the vessel is thoroughly "scraped, scrubbed, and fumigated" with *chlorine.* In *nine days* she receives again her healthy passengers, great pains being taken to exclude every grade and shade of diarrhœa. The vessel reaches the port of New York without any more sickness. The passengers are detained on board the vessel, for want of other quarantine accommodations, many days, yet no cholera occurs among them. The vessel was purified, the passengers were purified; there may be a question about their baggage.

Another similar fact deserves a permanent record. The steamship *Helvetia* left Liverpool, bound to New York, April 27, of the present year, having many hundred emigrants on board. Before she had reached Queenstown, Ireland, cholera broke out among her passengers, and again first among emigrants from the continent, and a place where cholera was prevailing. The ship returned to Liverpool, the passengers are removed to hulks in the waters of that city, and are detained twenty-seven days. Meanwhile the vessel is inspected, (purified?) and furnished with a new outfit of bedding and blankets. Seven hundred of her former passengers are re-shipped, and she sails again from Liverpool on the 29th of May, reaches New York June 11, and there is no cholera on board, and none was known to follow her passengers after they were discharged from quarantine.

The term of quarantine at Halifax was dangerously short.—There were cases of cholera in the encampments certainly during several of the seven days the passengers were detained on land. The period of incubation, according to Dr. Baly, may be six days; according to Pettenkofer, twenty-one; then the diarrhœal stage is often of several days' duration. Our neighbors naturally wished to be rid of their unwelcome visitor as soon as the laws of hospitality would sanction a dismissal, and their experiment proved successful, but I think should not be considered as a precedent. The Liverpool time, twenty-seven days, is much safer.

The *Medical and Surgical Recorder*, June 30th, contains a letter which states that the ship *Agnes* was ready to sail from Antwerp for New York on the 13th of May last, when cholera suddenly broke out on board. The ship was detained till the 31st, when she sailed with her "well passengers," 235 in number. Fifty-six had died, and twenty-five were left in the hospital. On the breaking out of the disease, "the government officials and the marine commissioners took, with praiseworthy activity, all steps and enforced all regulations which were necessary to prevent the spread of the fearful epidemic." The sick were sent to a fort. Whether those not attacked were removed from the ship we are not told. The cholera did re-appear in this ship.

The Halifax pilot took a very dangerous position in relation to the *England*, being towed after the vessel under steam. Whatever may be the nature of the cholera poison, whether the result of decomposing excrements or a miasmatic product, with open ports it could hardly fail to fall into the wake of a moving steamer. He would have been far safer on deck. He doubtless in some way carried the disease into his family, perhaps through his own dejections, perhaps through a poison in his garments. The dates of the illness in the children are not given, further than that two had died and two were ill on the 20th of April. Then, regarding the family at Freshwater Bridge, there is a very strong probability that cholera in them was produced by a poison in the bedding they took from the water, although there is no direct proof that the bedding had belonged to the *England*. It was afloat in the harbor, and nobody knows any reason why any family or any other vessel should throw away bedding, while this vessel had the strongest motive for doing so. If these articles brought cholera with them, was it the dejections or a miasm that was the morbific agent?

The professional mind in this country and in England is inclining very strongly to the idea that the dejections are the real source of danger. In these lectures I have adopted the miasmatic theory, not because I have thought it could be demonstrated, but because it seemed to me to explain a larger number of acknowledged facts than any other. Most of these facts can undoubtedly be explained on the new German theory, and it may be that the further examination of it may give it the precedence. I have said that the next epidemic will probably enable the profession to decide what measure of confidence is to be given to the views of Profs. Thiersch and Pettenkofer. Already something has been acquired. If the family at Freshwater Bridge were poisoned by anything in the articles of bedding, that poison might as reasonably be charged to the dejections with which they were in all probability soiled, as to a miasm of which they might be the carrier. The discharges of the Halifax pilot may certainly have bred the disease in his family.

The *Medical Record*, June 15th, gives, from a German journal, some facts regarding the occurrence of cholera at Altenburg, Germany, last year. The readers of this journal will remember that that disease was supposed to have been caused by the dejections of a child who had been only, if at all, exposed to cholera, and who "died of debility." The mother and child had traveled nine days and nine nights, the latter followed by diarrhœa the whole time. They had passed by certain places in the vessel where cholera was raging. The mother was attacked with cholera three days after reaching Altenburg. Was it the child or the mother that brought the disease? If the child, as Fettenkofer believes, then a diarrhœa that ends in "debility" can produce the cholera. But the mother and child were probably exposed to the same external dangers.

There was an outbreak of cholera in or near Elizabeth, N. J., on the 19th of June of the present year, which was charged to the account of discharges of a diarrhœal character. No official account of this attack has yet appeared; but it is claimed that a person from one of the quarantined vessels in New York harbor, visited in a filthy lane, had diarrhœa, but not cholera, and that other persons using the same privy were the first to be attacked. Twenty-one, it is said, were stricken with it, of whom nine died. The mayor of Elizabeth caused the locality (Dutch lane) to be purified immediately, and in less than a week the plague was stayed. He writes, June 27th, "The reports in regard to cholera in Elizabeth have been greatly exaggerated. We have had but a few cases in a low, filthy, and sparsely populated locality, but at this time there are no cases, and there have been none for the last three days." "The affected district," he says, "has been thoroughly cleansed and disinfected, and the disease has entirely disappeared." This unusual result was the fruit of unusual decision on the part of the mayor. The good work was done at his own cost, without waiting for the, perhaps, tardy action of other branches of the government. Whether it is true or not that this disease had the origin here ascribed to it, it is claimed by Pettenkofer that the early diarrhœal discharges are no less dangerous than the "rice-water" discharges, and equally require disinfecting.

The medical officer of the Privy Council of England has issued his warning against drinking water that is in any degree tainted by house refuse or any other like kinds of filth, and breathing the air which is made foul with effluvia from the same sorts of impurities. He further announces his belief that all matters which cholera patients discharge from the stomach and bowels are infective. "*This is equally the case whether the patient suffers from the disease in its developed and alarming form, or from the slightest diarrhœa which the epidemic influence can produce.*" (Health Report, N. Y. Daily Times, August 11.)

In the same issue of the *Daily Times*, we find the following:— "Patrick Kelly died of cholera at Hunter's Point on the 1st of August. The premises were disinfected under the direction of a sanitary officer. A portion of the clothing used about the patient, after what was regarded as a thorough application of chloride of soda, was washed, and no harm came of it. The heavier bed-clothing was treated in a tub with the same agent and afterwards buried, with strict orders that it was not to be exhumed, except under his direction, when disinfectants should be re-applied. But on the 4th, a Mrs. Dunn was made partially drunk, and persuaded by Mrs. Kelly to dig up the clothes and wash them. This she did that evening and the next (Sunday) morning. She went home drunk at five o'clock, Sunday morning. At eight P. M. of the same day, she was attacked with cholera, and died at six P. M. the next day. She dwelt in a shanty far removed from any cholera, and the attack seems traceable to the clothing. Inspector

Trask, who reports this case, says that chloride of soda has no disinfecting power over cholera poison, and that only coal-tar can be trusted for complete disinfection, and in this opinion Dr. Harris concurs."—*Health Report.*

Statements like these deserve the fullest consideration. As I have already said, we may not reject the practical inferences of the German doctrine; and I am free to confess that, in the light of many facts which the present season has developed, I am half-inclined to adopt, not the refinements of Pettenkofer, but the more simple views of Thiersch. That the discharges are poisonous when first evacuated is disproved by all the considerations adduced to show that cholera is not contagious, as typhus or scarlet fever is. They are dangerous in consequence of some change that occurs in them after they are voided, if dangerous at all. On Thiersch's theory, I cannot account for the well known facts that the first cases in any locality are the most malignant, and that after a certain number of days the disease grows milder and the cases less numerous, till it ceases in that locality; in other words, when there is least of the cholera poison the disease is most virulent, and after twenty days or so, when the cases are most numerous and the sources of the poison most abundant, the disease assumes a less malignant type. But I will not argue this point. The privies in which cholera discharges are deposited appear to be dangerous; the soiled beds, bedding, and garments appear to become dangerous. Let them be treated as the enemies of men, and let theories wait on further observation and study.

One discovery, growing out of theory to be sure, but itself worth more than all theories, has been strikingly confirmed in numerous instances the present year—the power of certain chemicals, called disinfectants, aided by the removal of filth, to stop the progress of cholera in limited localities, as a house or portion of a street, and by inference in all houses and streets. It is difficult, perhaps impossible, to trace relations between individual cases in a large city; but once more we have the fact that cholera did not appear in New York till after infected vessels had arrived in our harbor. If we can trust the newspaper reports, the disease broke out among the troops on Hart's Island, Governor's Island, and on a transport carrying soldiers to Tybee Island, only after sending to these places, and by this ship, recruits newly arrived in emigrant vessels; but wherever it has appeared within the jurisdiction of the Board of Health, out of public institutions, it has been met with a promptness and vigor not unexpected from such men as constitute that Board and deserving the highest praise. For three and a half months there have been scattered cases, and a few times small groups of cases occurring here; but I believe I announce the literal truth when I say that it has been promptly driven out of every house it has entered. The officers of the Board are ready at all hours, with their disinfectants distributed in wagons, horses harnessed, and medical officers in waiting, to drive to and disinfect any and every house where the disease appears. It has been thus systematically pursued from its very first occurrence to this moment. It has not been finally hunted down, and probably will not be till near winter, because the disease is rapidly increasing in the commercial towns of Europe, and also in the interior districts of Germany from which emigrants are daily arriving. In the month of May it is reported that 40,000 emigrants were landed in New York. Notwithstanding the vigilance of the quarantine officers, some of these will carry cholera with them, either by protracted incubation, or unreported diarrhœa, or in their unpurified baggage, and it will break out anew among us. The present indications are that deaths from cholera on Manhattan Island during the year will not exceed 500, while the deaths from ordinary causes will be as heretofore 22,000 or more. This low mortality is due to the unwearied and successful efforts of the Board of Health to expel it from every new lodgment it makes, to purify the city, and admonish citizens of their personal and domestic obligations. Up to August 17th, I am officially informed, excluding the islands in the East River, the deaths from cholera in New York have been only 247.

The cholera has lately broken out with great violence in the charitable and penal institutions of Blackwell's Island, destroying in the workhouse two in every thirteen of its inmates in nine days. The following letter of Dr. Hamilton, giving an account of its arrest in this institution and its mitigation in the others (from the Health Report in the *Daily Times*,) is worth volumes of argument, and should be re-published in the medical journals at once:

No. 64 Madison Avenue,
New York, Friday, Aug. 10, 1866.

E. Harris, M. D., Corresponding Secretary M. B. H.:

Sir:—The first case of cholera occurred in the workhouse on the 28th of July, the last case on the 6th of August. The epidemic continued, therefore, nine days, during which period, of about 800 inmates, 123 died. I do not mention one case reported on the 8th of August, because, as I understand, the person was admitted only the night before; I do not think the disease was contracted in the workhouse.

You know the building very well. It is admirably constructed for the purposes for which it is designed, and, so far as my observation extends, it is always perfectly clean. Until now, the inmates have been as healthy as this class of people are usually fouud to be.

The explanation of the rapid propagation and fatality of the disease after it once had gained admission was believed to be mainly confinement and crowding. It was observed that the cholera was for several days exclusively among the women. The women had the smallest apartments, were most crowded in their cells, and, with few exceptions, were employed within the building in close contact with each other during the day. The men were employed mostly in the quarries, out doors.

On Wednesday, when the epidemic was at its height, the 1st of August, I gave my pledge to the Board of Commissioners and to Mr. Schultz, President of the Board of Health, in your presence, that I would drive the cholera from the workhouse in from three to five days. I said this in no spirit of boasting, but in simple reliance on the well known and established laws of hygiene. The Commissioners executed literally and promptly every order which was given by the Committee.

The epidemic began to decline from the day they were fully carried out, and on Monday last the pledge was redeemed. The following is a summary of the sanitary means adopted:

The inmates were distributed as far as the vacant places in the building would permit; the cell-doors were left open at night; the night-buckets were supplied with disinfectants and left outside; the women's cooking-rooms were converted into hospital wards, and the women were kept out of doors from morning until night; corn-meal and molasses were taken from the diet table; coffee, tea and vegetables were added; at night each inmate was required to take, whisky one ounce, water three ounces, tincture of capsicum fifteen drops. [These people are our city vagrants, and probably are habitually intemperate.] A variety of disinfectants were employed freely and constantly in every vessel and closet which received the excreta; even the excreta from the stomach were disinfected immediately after they were received into a vessel or fell upon the floor; stoves were placed in each hospital ward to insure a draught; all windows were kept open day and night; the clothing taken from cholera patients was sent directly to the boilers; a ward was establiehed for patients with the diarrhœa, and the value of this measure is shown by the fact that of the large number received into this ward only one died. It was difficult, however, to persuade these poor creatures to report themselves at this stage of the disease.

From the workhouse the cholera has spread to every other building on the island, except, I think, to the madhouse, the pavilion attached to the male almshouse, and the fever pavilion. In none, however, has it proved so fatal as in the workhouse.

The same sanitary measures have been adopted, with slight modifications, in each department, but they cannot be applied with so much vigor to the lunatic asylum, the almshouse, or the general hospital. These buildings are all crowded, and the inmates can not be scattered or turned out of doors; consequently, the cholera remains among them, but in a greatly mitigated form. In the penitentiary it remained but two days.

Connected with the almshouse, are two well constructed pavilions, lying side by side, separated by only a few feet and a brick wall ten or twelve feet high. One is occupied by feeble old men, the other by the same class of old women. The only point of difference which I can discover is that at the time of the outbreak of the cholera the male pavilion contained only sixty-two persons, while the female contained ninety-nine. In the first there has not been one case of cholera; in the second thirty-one have died.

Of fourteen house-physicians and surgeons employed in these several buildings, some of whom have been in constant attendance upon the sick, not one has suffered from the epidemic.

Very respectfully yours,

FRANK H. HAMILTON, M. D.

[*N. Y. Medical Record*—Paper by A. Clark, M. D.]

